# Removal Efficiency of Faecal Indicator Organisms, Nutrients and Heavy Metals from a Peri-Urban Wastewater Treatment Plant in Thohoyandou, Limpopo Province, South Africa

**DOI:** 10.3390/ijerph120707300

**Published:** 2015-06-29

**Authors:** Joshua N. Edokpayi, John O. Odiyo, Titus A. M. Msagati, Elizabeth O. Popoola

**Affiliations:** 1 Department of Hydrology and Water Resources, University of Venda, Private Bag X5050, Thohoyandou 0950, South Africa; E-Mail: john.odiyo@univen.ac.za; 2 College of Science, Engineering and Technology, Nanotechnology and Water Sustainability Research Unit, Florida Science Campus, University of South Africa, 1710 Roodepoort, Johannesburg, South Africa; E-Mail: msagatam@unisa.ac.za; 3 Department of Chemical Sciences, Yaba College of Technology, P. M. B. 2011 Yaba, Lagos, Nigeria; E-Mail: seunliz27@yahoo.com

**Keywords:** *E. coli*, effluents, health, influent, impacts, treatment, wastewater

## Abstract

Wastewater treatment facilities are known sources of fresh water pollution. This study was carried out from January to June 2014 to assess the reduction efficiency of some selected contaminants in the Thohoyandou wastewater treatment plant (WWTP). The pH and electrical conductivity of the effluent fell within the South African wastewater discharge guidelines. The WWTP showed the chemical oxygen demand reduction efficiency required by the Department of Water Affairs (DWA) guidelines of 75 mg/L for the months of April and June, although it was below this standard in March and May. Free chlorine concentration varied between 0.26–0.96 mg/L and exceeded the DWA guideline value of 0.25 mg/L. The concentration of nitrate-nitrogen (NO_3_^−^ N) in the influent and effluent varied between 0.499–2.31 mg/L and 7.545–19.413 mg/L, respectively. The concentration of NO_3_^−^ N in the effluent complied with DWA effluent discharge standard of 15 mg/L, except in April and May. Phosphate concentrations in the influent and effluent were in the ranges of 0.552–42.646 mg/L and 1.572–32.554 mg/L, respectively. The WWTP showed reduction efficiencies of *E. coli* and *Enterococci* during some sampling periods but the level found in the effluent exceeded the recommended guideline value of 1000 cfu/100 mL for faecal indicator organisms in wastewater effluents. Consistent removal efficiencies were observed for Al (32–74%), Fe (7–32%) and Zn (24–94%) in most of the sampling months. In conclusion, the Thohoyandou WWTP is inefficient in treating wastewater to the acceptable quality before discharge.

## 1. Introduction

The principal objective of wastewater treatment is generally to allow domestic and industrial effluents to be disposed without endangering human health or cause unacceptable damage to the natural environment [1]. In many countries, urbanization and population are growing at an unprecedented rate and such development is often unbalanced with sufficient municipal expenditures devoted to high profile infrastructure for waste disposal and management coming well down in the list of priorities in terms of allocation of funds [2]. Effective collection and treatment of wastewater is a global problem, but it is more critical in the majority of developing countries. Due to the high costs involved in wastewater treatment, many of the underdeveloped and developing nations of the world have not been able to treat their wastewater to appropriate and satisfactory levels and continue to use it in agriculture with deleterious long-term effects on soil, groundwater and human health [3]. 

Mekala *et al.* [4] noted that in most developing countries, lack of sufficient funds, the high treatment costs of conventional treatment systems and rapid increases in wastewater generation that exceed the current capacities of the wastewater treatment plants (WWTPs), results in a poor percentage of wastewater undergoing correct primary/secondary treatment. Consequently, farmers whose lands are along these water bodies often channel the partially treated or untreated wastewater that is released into the rivers and lakes for irrigation [5]. It has been argued that although provincial/state governments receive grants to cover the capital costs of wastewater treatment plants, they allocate insufficient money to correctly operate and maintain these treatment plants and as a result the very need for their establishment fails [4].

In South Africa, the wastewater infrastructure is well developed in most urban areas as opposed to rural areas where the infrastructure is either poorly developed or non-existent [6]. The declining state of municipal wastewater treatment infrastructure in South Africa is one of the largest contributors to health problems in poor communities [7]. Since 2004, various studies and research have noted that up to 70% of municipal waste treatment facilities in South Africa is at the point of collapse for lack of proper maintenance and expansion [8,9]. This finding corroborates the report by Saving Water South Africa [10] that less than half of South African’s WWTPs treat effluents they receive to a safe and acceptable level. Experts on wastewater treatment in South Africa have raised concerns over the inability of the country’s local authorities to cope with the constant increasing demand for effective sewage treatment systems [11,12]. 

With sewage pollution, rivers and dams become eutrophic and algal blooms could develop, rendering the water difficult to treat with normal water treatment methods and consequently impacting the water with an unpleasant taste and odour [13]. The use of inadequately treated wastewater for irrigation in Malamulele area of Limpopo Province in South Africa has been linked to diarrhea and parasitic infection among irrigation farmers and their families with possible risks on the consumers that consume raw vegetables from those farms [5]. Momba *et al.* [14] studied the impact of inadequate wastewater treatment on the receiving water bodies in the Buffalo City and Nkokonbe Municipalities of the Eastern Cape Province and their results showed non-compliance with the Department of Water Affairs effluent discharge guidelines (DWA). A report from All Africa news had shown that only 7% of wastewater treatment systems in South Africa meet international standards [15]. Ogola *et al.* [16] believed that these findings could be due to inadequate investment in wastewater treatment infrastructure, shortage of skilled manpower, poor planning or corruption. 

Wastewater needs to be adequately treated prior to disposal or reuse in order to protect receiving water bodies from contamination. The discharge of poorly treated wastewater usually affects water users downstream and groundwater [17,18]. The health risks from inadequately treated wastewater usually come from microbial pathogens, nutrient loads, heavy metals and some organic chemicals [19]. Bacteria species are the most common pathogens usually encountered in treated wastewater and cause several infections and diseases particularly to young, pregnant, immune-compromised and aged people [20]. Very few studies have been reported that look into wastewater treatment plants on a broader scope as this study; heavy metals component is largely overlooked and not included in routine wastewater monitoring. This study is therefore aimed at assessing the phase and overall efficiency of Thohoyandou wastewater treatment plant that serves peri-urban and rural communities around it. 

## 2. Experimental Section 

### 2.1. Plant Description and Study Site

**Figure 1:**
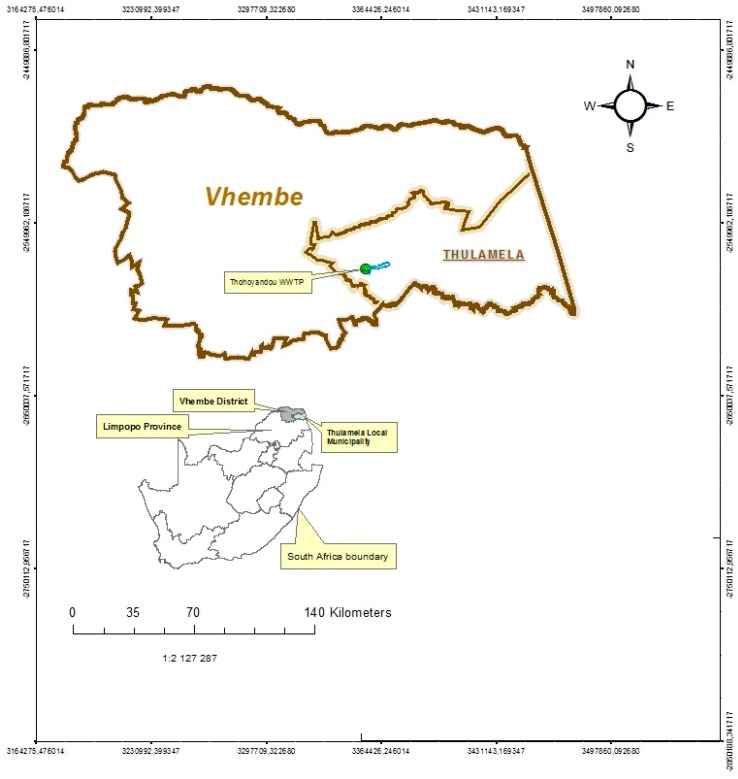
Map of the location of the WWTP in Muledane, Thohoyandou.

**Figure 2:**
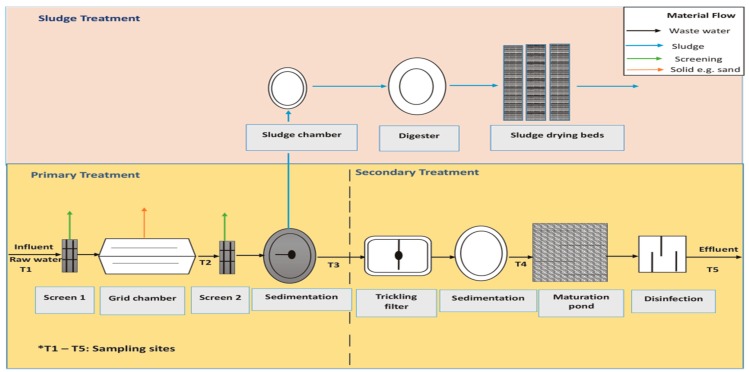
Basic processes in Thohoyandou WWTP.

The WWTP under study is located in the Muledane area of Thohoyandou in Vhembe District, Limpopo Province, South Africa, with geographical coordinates of 30°28'28''E and 23°0'13''S (Figure 1). The plant receives domestic sewage from residential areas, light industrial wastewater, wastewater from the University of Venda, clinics and agricultural firms, as well as run-off water from peri-urban areas and villages around Thohoyandou, Shayandima and Manini. The treatment is based on biological filters [21]. The design capacity of the plant at inception in 1981 was 3 megaliters of wastewater per day; due to the population increase the plant capacity was subsequently extended to 6 megaliters of wastewater per day. The WWTP is currently overstressed, as it now receives about 13 megaliters of wastewater per day [21]. The effluent is discharged to the Mvudi River, which is used for domestic, recreational and agricultural purposes, and as a major source of water to the Nadoni Dam. Figure 2 shows the basic unit operations at the plant.

### 2.2. Sample Collection and Field Measurements

Wastewater samples were collected from five points in the treatment plant designated as T_1_–T_5_ in Figure 2. The samples for metals and non-metals determination were placed in polyethylene bottles that had been pre-washed with non-ionic detergents and rinsed with distilled water, while those for microbiological analysis were placed in new polyethylene bottles in sterile condition. The sample bottles were rinsed with the water samples from the treatment plant before collection. Three sets of samples were collected from each sampling point; a set of the samples intended for total trace metal analysis was acidified using 5 drops of concentrated nitric acid. The others two sets were used for anions and microbiological analyses. Field measurements of pH and EC were carried out using a 340i Multimeter (WTW, Weilheim, Germany) while turbidity was measured using a TB 200 portable turbidimeter (Orbeco Hellige, Sarasota, FL, USA). All samples were transported in an ice chest to the laboratory. 

### 2.3. Chemical Oxygen Demand (COD) Measurement 

Measurement of COD was performed using COD test Kits on a Spectroquant photometer (Merck, Johannesburg, South Africa) equipped with a thermoreactor. Two milliliters of the sample was carefully run from a pipette into the reaction cell. Prior to this, the content of the reaction cell was homogenized by swirling for one minute. The sample and the content of the reaction cell were vigorously mixed and then transferred to the thermoreactor where the cell was heated up to 148 °C for 120 minutes. The cell was subsequently removed from the thermoreactor and measured in a photometer after cooling for 40 min.

### 2.4. Analysis of Microbiological Parameters 

*E*. *coli* and *Enterococci* counts were analysed in each sample as described in the method recommended by the American Public Health Agency [22]. Sample aliquots of 100 mL were filtered through a 0.45 µm pore size, 47 mm diameter filter membrane (Millipore, Johannesburg, South Africa). The filters were placed in a previously prepared mFC and mEnterococcus agar (Acumedia, Pretoria, South Africa) contained in petri dishes which are suitable for culturing the target bacteria species. This was subsequently placed in an incubator at 37 °C and 45 °C for 24 and 48 hours incubation, respectively, for the total and fecal coliforms. The samples were analysed in duplicate and recorded as colony forming unit per 100 mL. 

### 2.5. Anion Analysis

Fluoride, chloride, and phosphate were analysed using a 850 professional IC (Metrohm, Johannesburg, South Africa) connected to an autosampler. Prior to analysis, four calibration standards of 1 mg/L, 5 mg/L, 10 mg/L and 20 mg/L were prepared by serial dilution using a 100 mg/L multi element stock solution purchased from Merck-Millipore (Johannesburg, South Africa). The samples were filtered through 0.45 micron syringe filter and placed in an autosampler. A combination of sodium carbonate and sodium bicarbonate were used as the eluent. The eluent was degassed before introduction to the IC system. Sulfuric acid (50 mmol/L) was used as the suppressant with a flow rate of 0.5 mL/min. Free chlorine and nitrate nitrogen were determined using a HACH potable colorimeter and DR 3900 Spectrophotometer (HACH, Johannesburg, South Africa). 

### 2.6. Digestion and Analysis of Water Samples 

The method recommended by US EPA [23] was used for the digestion of water samples: 3 mL of concentrated HNO_3_ was added to 50 mL of the sample in a beaker. The beaker was covered with a ribbed watch glass. The solution was placed on a hot plate and heated in a fume cupboard without boiling to less than 5 mL. The sample was allowed to cool after which 5 mL of concentrated nitric acid was added. The beaker was covered with a non-ribbed watch glass and returned to the hot plate. The temperature of the hot plate was increased so that a gentle reflux action occurs. The heating continued, until digestion was complete and allowed to cool. Ten milliliters of 1:1 HCl and 15 mL of de-ionise water were subsequently added. The resulting solution was heated for an additional 15 minutes. The beaker walls and watch glass were rinsed with de-ionised water and subsequently filtered using a Whatman No 1 filter paper (with diameter of 90 mm). The filtrate was transferred to a 100 mL volumetric flask and made up to 100 mL with distilled water.

### 2.7. Analysis of Heavy Metals

Heavy metals in the digested samples were analysed using a ICP-OES instrument (ICAP 6500 DUO, Thermo Scientific, Johannesburg, South Africa). The operating conditions employed for ICP-OES determination were 1150 W RF power, 0.5 L min^−1^ auxiliary flow, 0.7 L min^−1^ nebulizer flow, 1.5 mL min^−1^ sample uptake rate, plasma stabilization time of 10 mins and 12 L/min coolant gas flow. Duo view was used for all the metal determination, while 2-point background correction and three replicates were used to measure the analytical signal. The emission intensities were obtained for the most sensitive lines free of spectral interference. The calibration standards were prepared by diluting the stock multi-elemental standard solution (100 mg/L). The method detection limit (MDL) for each metal was obtained by US EPA method 200.7 [24].

### 2.8. Compliance Study and Calculation of Percentage Reduction Efficiencies

The Department of Water Affairs of South Africa (DWA) and the Environment Canada guidelines were used as benchmarks to evaluate the acceptability of the effluent from the WWTP due to the prevailing environmental conditions in South Africa and the scope of parameters stated in the guidelines. The reduction efficiencies of the various parameters were calculated by Equation (1):

(1) $$\text{Reduction efficiency}=\frac{\text{concentration (level) in the influent}-\text{concentration (level) in the effluent}}{\text{concentration or level in the influent}}\times 100$$

## 3. Results and Discussion

### 3.1. Effect of Sample pH

The pH of wastewater as it passes through the WWTP is a very important factor for heavy metal and organic compound removal [25]. Precipitation aided by the addition of chemicals is the most widely used method for trace metal removal from wastewater. This is because most metals are easily precipitated as insoluble solids at alkaline pH [26]. The pH of the influent during the sampling period was in the range of 6.9–7.7 (Table 1). This pH range is not suitable to precipitate most metals present in the wastewater [26]. A general trend observed in the treatment plant is a small change in pH after each process. This could be due to lack of addition of Ca(OH)_2_ or insufficient addition of lime to the wastewater, which could also point to the inefficient management of the plant. pH control is critical and not limited to the precipitation of metals alone [27]. The pH range observed is, however, suitable for the breakdown of organic material in the wastewater to stable inorganic substances by bacterial species [28]. Rigorous precision of pH control is often required for treatment. Although the plant has an automated system for the addition of Ca(OH)_2_, the quantity of water entering per time is not properly monitored and the pH of the raw water is also not recorded. This could be the reason why the plant was not efficient in the removal of some metals. The pH of the effluent (7.1–7.5) was within the recommended guidelines by the Departmt of Water Affairs [29] and Environment Canada for effluent disposal unto natural water or streams [30].

### 3.2. Electrical Conductivity

Electrical conductivity (EC) gives an estimate of the presence of ionic substances in water. It is often used as a surrogate measure of total dissolved solids present in the water and wastewater [29]. It also gives information about the salinity of water and its suitability for use in irrigation. The influent had a low concentration of ions, which varied between 32.3–136.8 mS/m (Table 1). EC values of the effluent was greater than the influent in January and February, which were periods of heavy rainfall. The result obtained after primary sedimentation is unexpected because an increase in the levels of ions were determined instead of a decrease except in January but a decrease was observed after secondary sedimentation. Looking at the values obtained in Table 1, the efficiency of each unit process is questionable. Percentage reduction of ions was very low, varying between 8.4–34%. The EC values were generally within the effluent discharge limit of 150 mS/m [29].

**Table 1:** Levels of physico-chemical parameters through the major stages of the Thohoyandou WWTP.

Parameters	Influent	After screening and lime addition	After primary sedimentation	After secondary sedimentation	Effluent	% Reduction
pH _January_	7.1	7.0	6.9	7.4	7.2	NA
pH _February_	7.6	7.4	7.3	7.2	7.1	NA
pH _March_	7.7	7.3	7.2	7.2	7.2	NA
pH _April_	6.9	6.8	6.9	6.8	7.2	NA
pH _May_	7.2	7.3	7.3	7.5	7.4	NA
pH _June_	7.3	7.5	7.4	7.4	7.5	NA
EC _January_	32.3	30.7	29.1	32.3	34.0	-
EC _February_	35.7	37.0	43.0	35.1	35.8	-
EC _March_	44.2	44.9	48.3	42.8	39.7	10
EC _April_	60.4	63.4	66.7	46.2	39.9	34
EC _May_	58.2	58.7	61.1	47.9	43.1	26
EC _June_	136.8	139.2	154.8	121.3	125.3	8
T _January_	56.7	48.6	85.8	6.2	4.8	92
T _February_	52.9	49.3	60.1	14.0	7.2	86
T _March_	90.5	133.5	73.0	24	10.1	89
T _April_	114.9	125.5	68.2	14.8	6.4	94
T _May_	180.8	134	88.5	30.2	14.6	92
T _June_	62.6	110.6	35.7	6.0	4.3	93

EC = electrical conductivity (mS/m), T = turbidity (NTU), NA = Not applicable.

### 3.3. Turbidity

Turbidity tests are very crucial in wastewater treatment as they indicate the quality of wastewater in respect to colloidal and residual suspended matter [31]. The measurement is based on the comparison of the intensity of light scattered by a sample with respect to the light scattered by a reference material under the same conditions. The turbidity values for the influent were in the range 52.9–180.8 NTU (Table 1). This indicates the presence of a large quantity of suspended solids. After screening and primary sedimentation, the turbidity values were expected to reduce abruptly but this was not the case. A great reduction in turbidity was however observed after secondary sedimentation. An overall reduction efficiency of 86–94% was observed during the study period. The effluent had turbidity values in the range of 4.3–14.6 NTU (Table 1). The values of turbidity obtained in this study are comparable to 3.6–9.6 NTU reported by Igbinosa and Okoh [32] from the effluent of a wastewater treatment plant in Eastern Cape of South Africa, within 16–34% deviation. The Department of Water Affairs of South Africa currently has no wastewater discharge guideline for turbidity.

### 3.4. Chemical Oxygen Demand (COD)

**Figure 3:**
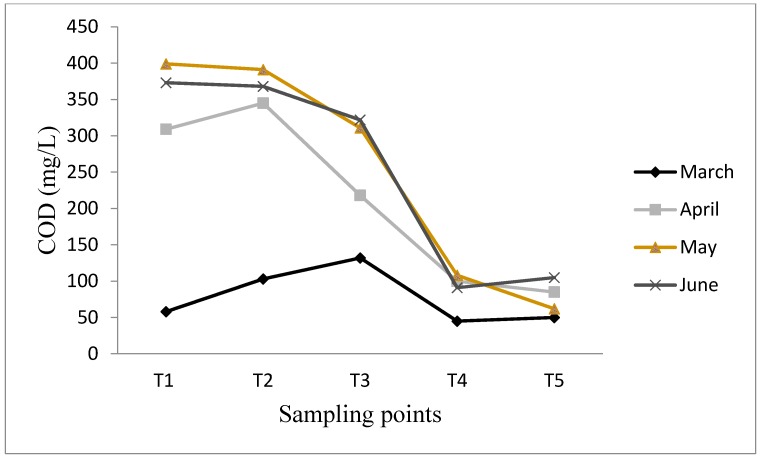
COD concentrations in different sampling points in Thohoyandou WWTP.

Chemical Oxygen Demand measurement in wastewater determines the oxygen equivalent of organic substances which can be easily oxidized chemically. The influent concentration of COD ranged between 58–399 mg/L (Figure 3). The expected trend of COD reduction after each stage of wastewater treatment was mostly not discernible during the wet season (January–March). For example in March, there was no percentage reduction in COD after primary sedimentation; this may be due to short residence time of the wastewater in the treatment plant as a result of the influent volume exceeding the design capacity of the plant due to heavy rains. However, 37%, 21% and 40% reduction in COD (as calculated from Equation (1) and Figure 3) were observed after primary sedimentation in April-June, respectively. Similarly, 66%, 54%, 65% and 72% reduction in COD were recorded after secondary sedimentation from March–June, respectively. This clearly shows that the wastewater treatment plant is effective in reducing the concentration of organic molecules present in the influent. Overall efficiencies of 14%, 73%, 85% and 72% were observed in March-June, respectively. A very low reduction in COD was observed in March (14%) as compared to April-June (72–85%); this may be attributed to rainfall events in March that leads to increased quantity of wastewater. This will reduce the residence time of the wastewater in the treatment plant because the design capacity of the plant was overstretched. 

The effluent COD concentrations were in the range of 50–105 mg/L (Figure 3). Although COD concentrations were reduced beyond 50%, except in March, the concentrations were within the recommended guideline value of 75 mg/L by DWA for the discharge of wastewater effluents into a water resource system for the months of March and May, but exceeded this value in April and June. Igbinosa and Okoh [32] recorded COD effluents concentrations in the range of 36.82–238 mg/L from a wastewater treatment plant in the East of Alice town of Eastern Cape Province, South Africa. The release of effluent with high COD concentration continuously into a water course is undesirable since it has negative effects on the quality of water in the receiving river and can cause harm to fish and other benthic organisms further downstream [32,33].

### 3.5. Concentrations of Anions in the Samples

Although some anions like fluorides, chlorides and sulphates are not usually determined in the effluent of WWTP, their concentrations are important because they could contribute significantly to the contamination of the receiving stream. Low fluoride concentrations were noted in the influent and the effluents during the sampling months. The influent had F^−^ concentrations in the range of 0.152–1.269 mg/L while the effluent concentration varied between 0.102 and 1.562 mg/L (Table 2). An unusual high concentration of F^‒^ was observed in the influent and effluent in June (1.269 mg/L) and April (1.562 mg/L), respectively. The fluoride concentrations in the effluent did not exceed the DWA guideline value of 1 mg/L, except in April (1.562 mg/L) [34]. The reason for this observation is unclear. The WWTP only recorded a percentage reduction of F^‒^ in January (5.92%), February (20.1%) and June (92%). The concentration of F^‒^ in the effluent is of no threat to the receiving water except in the month of April.

**Table 2:** Concentrations of anions through major stages of the Thohoyandou WWTP.

Anion concentration (mg/L)	Influent	After screening and lime addition	After primary sedimentation	After secondary sedimentation	Effluent	% Reduction
Fluoride _January_	0.152	0.140	0.146	0.145	0.143	6
Fluoride _February_	0.244	0.030	0.031	0.143	0.195	20
Fluoride _March_	0.181	0.186	0.030	0.186	0.181	-
Fluoride _April_	0.167	1.333	1.155	1.484	1.562	-
Fluoride _May_	0.051	0.051	0.066	0.063	0.344	-
Fluoride _June_	1.269	0.193	0.182	0.053	0.102	92
Chloride _January_	18.933	15.090	13.584	14.035	15.293	19
Chloride _February_	25.963	25.247	28.770	25.085	25.570	2
Chloride _March_	38.870	38.650	36.220	31.880	28.020	28
Chloride _April_	50.765	47.659	43.478	53.755	56.524	-
Chloride _May_	41.024	28.534	29.092	27.008	31.151	24
Chloride _June_	51.971	60.365	63.066	57.318	49.116	6
NO_3_^-^ as N _January_	0.674	0.663	0.910	14.089	14.078	-
NO_3_^-^ as N _February_	0.994	0.962	1.748	13.293	7.545	-
NO_3_^-^ as N _March_	2.310	3.230	1.100	17.030	12.010	-
NO_3_^-^ as N _April_	0.546	2.668	3.651	8.5170	16.398	-
NO_3_^-^ as N _May_	0.499	0.787	7.211	80.961	19.413	-
NO_3_^-^ as N _June_	0.978	1.220	0.122	146.72	12.367	-
PO_4_^3-^ as P _January_	0.552	0.685	0.665	1.387	1.572	-
PO_4_^3-^ as P _February_	1.220	1.240	2.970	2.700	2.220	-
PO_4_^3-^ as P _March_	2.330	1.960	2.730	3.270	2.940	-
PO_4_^3-^ as P _April_	4.388	5.379	9.136	3.745	4.836	-
PO_4_^3-^ as P _May_	4.265	4.085	5.295	10.489	3.255	24
PO_4_^3-^ as P _June_	2.635	2.897	2.920	2.947	2.500	5

Chloride concentration in the influent varied in each sampling month. The concentrations varied between 18.93–51.97 mg/L (Table 2). There was a slight reduction in Cl^‒^ concentration in each month except for April. Reduction efficiencies of 19%, 2%, 28%, 24% and 6% were observed in January, February, March, May and June, respectively. No sequential reduction of chloride ions was recorded as wastewater passed from one treatment stage to another. The concentrations of Cl^‒^ in the effluent were in the range 15.29–56.52 mg/L. These concentrations are of no threat to the receiving water resource. There is no guideline value of Cl^‒^ in wastewater effluent but the concentration found is within the recommended guideline of 200 mg/L for chloride in domestic water. The concentration of free chlorine in the effluent during the study period ranged from 0.26–0.63 mg/L and exceeded the recommended guideline of DWA (0.25 mg/L) [34].

Table 2 shows the range of nitrate nitrogen (NO_3_^−^ as N) concentrations throughout the wastewater treatment process. The WWTP showed no percentage reduction of NO_3_^−^ as N throughout the sampling period as the concentration of the effluent was greater than the influent. The possible reason for the higher nitrate concentration observed in the effluent than influent could be due to incomplete denitrification caused by insufficient organic carbon required for respiration by denitrifying bacteria [35,36]. Breisha [37] reported that denitrification process are often slow and several carbon sources are usually added to wastewater to aid complete denitrification. Some of the most common carbon sources reported are methanol, ethanol and acetic acids [36,37,38]. In addition, the success of denitrification process is depended on several other parameters such as the type and concentration of carbon source, temperature, pH among others [35,37]. The effluent concentration of NO_3_^−^ as N complied with the stipulated guideline of 15 mg/L [29] in January (14.078 mg/L), February (7.545 mg/L), March (12.01 mg/L) and June (12.367 mg/L). Other sampling months exceeded the guideline. All the NO_3_^−^ as N concentrations obtained were higher than the Environment Canada effluent guideline of 1 mg/L, which is more stringent than that of South Africa [30]. 

Nitrate in wastewater must be regulated due to the known negative impact it can cause in the receiving watershed [14,32]. A concentration of NO_3_^−^ as N as low as 1 mg/L has been implicated as being capable of inducing eutrouphication, which affects aquatic organisms and thus reduces biodiversity, renders the water unfit for recreational purposes and leads to offensive odours that affect people living very close to the water resource [14].

Phosphate concentrations in the influent were generally higher when compared to NO_3_^−^ as N nitrate concentrations except in the month of January and were in the range of 0.552–4.388 mg/L (Table 2). However, the effluent concentrations of PO_4_^3−^ as P (1.572–4.836 mg/L) were lower than the effluent concentrations of NO_3_^−^ as N throughout the sampling periods. The effluent concentration complied with the recommended guideline value of DWA for wastewater discharge. The WWTP showed reduction efficiencies of PO_4_^3−^ as P only for the months of May (24%) and June (5%). This shows that the WWTP is partially effective in reducing phosphates ions from wastewater. High concentration of phosphate is known to accelerate algal growth causing eutrouphication, which consequently leads to reduction in dissolved oxygen and loss of some aquatic life forms [39,40,41]. A concentration of phosphate greater than 5 µg/L can cause unwanted algal growth in surface waters since it is a limiting reagent in natural waters [42].

### 3.6. Microbiological Test

**Figure 4:**
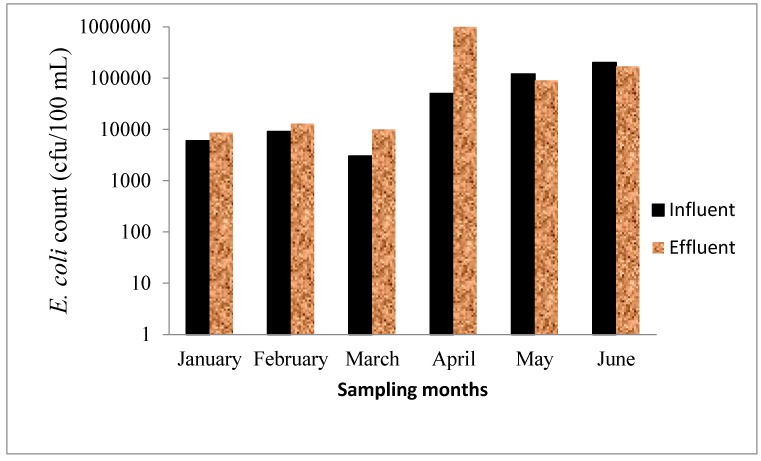
*E. coli* count in the influent and effluent of Thohoyandou WWTP.

**Figure 5:**
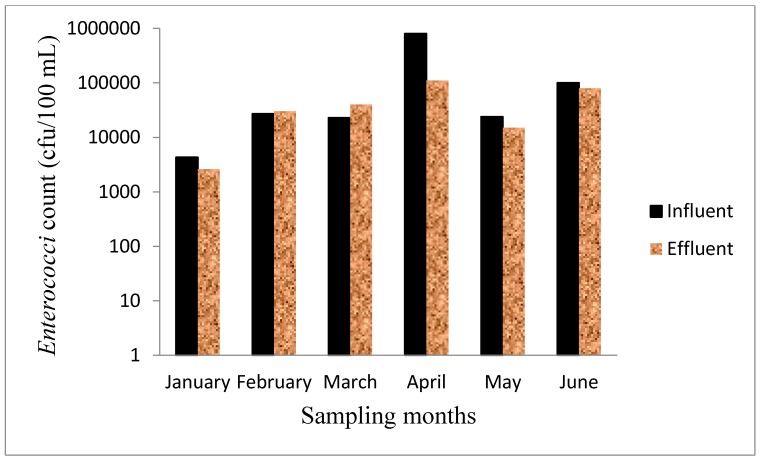
*Enterococci* count in the influent and effluent of Thohoyandou WWTP.

The microbial content in wastewater is a major threat to public health [43,44]. DWA and Environment Canada have fixed guideline values for faecal coliforms in wastewater effluent as 1000 cfu/100 mL and 100 cfu/100 mL, respectively [29,30]. Any count higher than any of these is considered unsafe and could pose environmental and health risk to the user of the river that receive such effluents. In this study, *E. coli* and *Enterococci* were chosen as most suitable faecal indicator organisms owing to their wide acceptance as such in different parts of the world [45,46]. The levels of *E. coli* and *Enterococci* varied in each sampling period. *E. coli* in the influent and effluent ranged from 6 × 10^3^–2 × 10^6^ cfu/100 mL and 8 × 10^3^–1 × 10^6^ cfu/100 mL, respectively (Figure 4) while *Enterococci* were in the range of 4 × 10^3^–8 × 10^5^ cfu/100 mL and 2.6 × 10^3^ cfu/100 mL and 1.1 × 10^5^ cfu/100 mL (Figure 5). The *E. coli* count in the wastewater was generally higher than that of *Enterococci*. No percentage removal of *E. coli* was observed for the months of January–April because the effluent counts were higher than the influent. However, 25% and 15% reduction efficiencies were determined in May and June, respectively. Similarly, *Enterococci* levels were higher in the effluent than the influent for the months of February and March, although, 40%, 86%, 38% and 20% reductions efficiencies were observed in January, April, May and June, respectively. The observed reduction in *E. coli* and *Enterococci* levels did not however meet the recommended guidelines of DWA for the discharge of wastewater effluents. The results obtained clearly indicate improper disinfection processes in the WWTP which is believed to be responsible for the high levels of faecal indicator organisms in the effluent. Thohoyandou WWTP is increasingly unable to cope with the quantity of wastewater it receives. There is lack of data from the WWTP showing the record of total or faecal coliform test performed on the effluent before discharge. There is no way the managers of the plant can ascertain the suitability for the disposal of effluents without performing these basic tests. The counts of *E. coli* and *Enterococci* in the effluent exceeded the DWA standard of 1 × 10^3^ cfu/100 mL for faecal coliform in wastewater effluents. The level obtained is very high and can cause acute to severe infections and diseases for those who depend on Mvudi River for domestic, irrigation and recreational purposes. 

### 3.7. Metals in the Wastewater Samples

The concentrations of metals are often not part of the routine tests usually carried out in a WWTP but due to their persistence, toxicity and bioaccumulation tendencies, it is desirable to determine their levels in wastewater effluents [18]. 

The discharge of metal-rich effluents is undesirable as it can lead to increased salinity of the receiving streams, negatively affect seaweeds, benthic organisms including fish species and zooplankton and can bio-accumulate in them to high concentrations such that humans that feed on them could be at risk [39,47]. The MDL for each metal ranged from 1 µg/L (Pb) to 25 µg/L (Al). The concentrations of Al in the influent varied from 1.60–3.90 mg/L (Table 3). 

The WWTP was able to reduce Al concentrations for each of the sampling months except January. The percentage removal was in the range of 32–74%. The concentration of Al in the effluent varied from 0.50–2.48 mg/L. The Department of Water Affairs does not have any guideline value for Al in wastewater effluent at present but 0.03 mg/L has been reported as the future guideline value [29]. The concentrations of Al in the effluent exceeded this latter guideline value for each of the sampling months but complied with the Environment Canada [30] value of 2.0 mg/L, except for January (2.48 mg/L) and May (2.13 mg/L).

The influent concentrations of Fe were in the range of 0.74–1.37 mg/L as presented in Table 3. The WWTP showed no removal efficiency for the months of January and June because its concentration was higher in the effluent than in the influent. The effluent concentrations of Fe varied between 0.49–1.33 mg/L and showed 35%, 18%, 5% and 7%, removal efficiencies from February–May. These values were beyond the DWA guideline of 0.3 mg/L for Fe in wastewater effluent for each of the sampling months but were within the Environment Canada guideline of 2.0 mg/L [29,30]. The DWA guideline value for Zn in wastewater effluent is 0.1 mg/L [29]. The concentration of Zn in the wastewater effluent exceeded this value in January (0.22 mg/L) and May (0.20 mg/L) but fell within the guideline value in other sampling months. Removal efficiencies were calculated for all the sampling months except January and were in the range of 27–94%.

**Table 3:** Heavy metals concentrations through major stages of the Thohoyandou WWTP.

Heavy metals concentration (mg/L)	Influent	After screening and lime addition	After primary sedimentation	After secondary sedimentation	Effluent	% Reduction
Al _January_	1.604	1.487	1.669	1.168	2.483	-
Al _February_	1.915	0.427	0.367	0.627	0.501	74
Al _March_	2.271	1.931	2.602	1.259	0.739	68
Al _April_	3.903	4.296	3.283	1.419	1.780	54
Al _May_	3.833	2.974	3.316	3.418	2.126	45
Al _June_	2.522	6.037	2.184	1.409	1.706	32
Fe _January_	1.129	0.920	1.073	1.282	1.329	-
Fe _February_	0.744	0.635	0.537	0.522	0.485	35
Fe _March_	0.916	0.801	0.951	0.831	0.746	18
Fe _April_	0.815	1.016	1.034	0.885	0.772	5
Fe _May_	1.374	1.116	1.134	3.401	1.284	7
Fe _June_	0.760	2.160	0.624	0.530	0.843	-
Zn _January_	0.121	0.091	0.121	0.146	0.217	-
Zn _February_	0.071	0.060	0.068	0.045	0.052	27
Zn _March_	0.091	0.097	0.099	0.105	0.051	44
Zn _April_	0.130	0.109	0.129	0.043	0.072	45
Zn _May_	0.344	0.245	0.193	0.126	0.202	41
Zn _June_	0.776	0.068	0.085	0.032	0.046	94
Cr _January_	0.476	0.206	0.501	0.455	0.329	31
Cr _February_	0.221	0.265	0.250	0.248	0.241	-
Cr _March_	0.299	0.254	0.283	0.282	0.301	-
Cr _April_	0.035	0.018	0.050	0.038	0.043	-
Cr _May_	0.422	0.360	0.249	0.384	0.422	-
Cr _June_	0.030	0.037	0.033	0.012	0.030	-
Cu _January_	0.048	0.080	0.058	0.118	0.267	-
Cu _February_	0.017	0.036	0.045	0.014	0.021	-
Cu _March_	0.032	0.025	0.031	0.030	0.029	9
Cu _April_	0.048	0.070	0.061	0.041	0.087	-
Cu _May_	0.055	0.042	0.055	0.066	0.035	36
Cu _June_	0.029	0.125	0.040	0.050	0.068	-
Mn _January_	0.098	0.045	0.091	0.068	0.067	32
Mn _February_	0.303	0.293	0.246	0.043	0.042	86
Mn _March_	0.225	0.197	0.235	0.251	0.227	-
Mn _April_	0.280	0.418	0.278	0.497	0.345	-
Mn _May_	0.276	0.221	0.265	0.331	0.899	-
Mn _June_	0.167	0.582	0.251	0.213	0.277	-
Pb _January_	0.003	0.006	0.005	0.008	0.008	-
Pb _February_	ND	0.002	ND	ND	ND	-
Pb _March_	0.002	ND	0.001	0.002	ND	-
Pb _April_	0.072	0.022	0.030	0.029	0.042	42
Pb _May_	0.005	0.007	0.012	0.068	0.010	-
Pb _June_	0.011	0.014	0.008	0.001	0.011	-

ND: Not detected.

Table 3 shows the concentrations of Cr in the influent (0.03–0.476 mg/L). The WWTP was inefficient for chromium removal, except in the month of January where a 31% reduction was recorded. The effluent concentrations of Cr were higher than the influent for the rest of the months. The DWA [29] and Environment Canada [30] guideline value for Cr in wastewater effluent is 0.05 mg/L, which was exceeded during all the sampling months except for April (0.04 mg/L) and June (0.03 mg/L). Similarly, 9% and 36% reduction of Cu was calculated only in the months of March and May. The effluent concentrations (0.021–0.267 mg/L) were beyond the guideline value of 0.01 mg/L stipulated by DWA for wastewater effluent [29]. 

The WWTP only showed 32% and 86% Mn removal in the months of January and February but was inefficient for the other sampling months. The effluent concentrations of Mn were in the range 0.04–0.9 mg/L. The effluent concentrations in January and February were within the DWA guideline value of 0.1 mg/L [34], but exceeded it for the other sampling months. Low concentrations of Pb were determined both in the influent and the effluent. Lead concentration ranged from 0.003–0.072 mg/L and 0.008–0.042 mg/L in the influent and effluent respectively. Pb was not detected in the influent in February and was also not detected in the effluent in February–March. The concentration of Pb in the effluent exceeded the influent concentration for the months of January and May. Percentage reduction efficiency of 42% of Pb was observed in April, but for June the concentration was the same for both the influent and the effluent. The concentration of Pb in the effluent complied with the DWA guideline value of 0.01 mg/L [29] except for months of April and June.

In summary, Thohoyandou WWTP is therefore enriching some of the metals which could be the consequence of resolubilization of insoluble metallic hydroxides in the sludge back to the wastewater which is carried to the next stage of treatment. This result is unexpected and contrary to various findings recorded in literature that noted a gradual reduction in metal concentrations throughout the WWTP [48,49,50,51,52]. However, selective reduction of Al, Fe and Zn was observed when compared to other metals. This can be attributed to the selective adsorption of these metals onto inorganic particles and living cells present in the wastewater [48,53]. Chipasa [48] noted that heavy metal removal efficiency is affected by a number of factors which include pH, initial metal concentration, composition of the wastewater, operating conditions and the type of metal specie present. pH plays an important role in metal removal efficiency and the solubility of metals vary at different pH values making it difficult to predict their levels at any given to time also owing to the complexity of wastewater composition [48].

Correlation studies were performed on the data obtained for the various parameters in the effluent from the WWTP. pH, EC and turbidity did not vary significantly with other parameters (*p* < 0.05). Cr exhibited a negative but significant correlation with Cl^−^ (r = −0.823, *p* < 0.05) and *Enterococci* levels (r = −0.908, *p* < 0.05). Nitrate only showed a positive correlation with Mn (r =0.823, *p* < 0.05). Phosphate correlated positively and significantly with Pb (r = 0.813, *p* < 0.01) and *E. coli* (*p* < 0.01). Al varied significantly with Fe (r = 0.887, *p* < 0.05) while Fe varied significantly with Zn (r = 0.927, *p* < 0.01). *E. coli* and *Enterococci* varied positively and significantly (r = 0.827, *p* < 0.05). Several positive insignificant correlations were also obtained between the parameters. Table 4 shows the results obtained from the correlation studies.

**Table 4:** Results from the correlation studies on the various parameters investigated in the effluent of the WWTP.

Variables	pH	EC	T	F	Cl	N	P	Al	Fe	Zn	Cr	Cu	Mn	Pb	E. coli	Ent
pH	1	0.801	0.113	−0.208	0.403	0.453	0.029	0.451	0.391	0.105	−0.182	−0.122	0.574	0.048	−0.063	0.178
EC		1	−0.385	−0.248	0.510	−0.093	−0.108	0.099	−0.089	−0.370	−0.597	−0.144	0.030	−0.001	−0.031	0.426
T			1	−0.054	−0.188	0.488	0.258	−0.066	0.249	0.311	0.690	−0.511	0.789	−0.201	−0.202	−0.370
F-				1	0.677	0.414	0.893*	0.155	−0.156	−0.143	−0.470	−0.042	0.174	0.943**	0.974**	0.706
Cl^−^					1	0.252	0.772	0.035	−0.330	−0.521	−0.823*	−0.361	0.245	0.759	0.800	0.954**
N						1	0.501	0.751	0.730	0.616	0.219	0.159	0.820*	0.517	0.376	0.051
P							1	0.007	−0.206	−0.268	−0.393	−0.399	0.453	0.813*	0.870*	0.731
Al								1	0.887*	0.776	0.096	0.694	0.381	0.399	0.189	−0.105
Fe									1	0.927**	0.517	0.617	0.472	0.022	−0.185	−0.489
Zn										1	0.677	0.614	0.394	−0.046	−0.235	−0.658
Cr											1	0.091	0.323	−0.580	−0.641	−0.908*
Cu												1	−0.354	0.123	−0.025	−0.286
Mn													1	0.210	0.126	−0.028
Pb														1	0.976**	0.749
*E. coli*															1	0.827*
ENT																1

EC is electrical conductivity, T is turbidity, N is nitrate nitrogen, P is phosphate, Ent is *enterococci*; * correlation is significant at 0.05 level (2-tailed); ** correlation is significant at 0.01 level (2-tailed).

This clearly indicates that the WWTP is not functioning properly as it should and the effluent it produces is of high risk to the receiving river. The absence of coagulant addition and appropriate chemicals for pH adjustment could be the reasons for the poor removal of metals with consequent enrichment during the treatment process. pH plays an important role in the solubility of metals and precipitation as insoluble compounds which can settle down during sedimentation stage. Most of the units operations in this WWTP are not functioning properly and hence do not meet the basic requirements of a wastewater treatment plant.

## 4. Conclusions 

Thohoyandou WWTP, which is typical of most WWTPs in rural and peri-urban areas of South Africa, is producing effluents of poor quality that could be injurious both to the environment and human health. Nitrate concentrations in the effluent for several months were very high and exceeded the guideline value and could lead to eutrouphication with its accompanying consequences. The WWTP was inefficient is reducing *E. coli* and *Enterococci* counts to the recommended levels and the removal of toxic metals was inadequate. A different trace metal concentration was determined for each of the sampling month. The major causes for these observations include poor investment in wastewater treatment in rural and semi urban areas of South Africa, small design capacity of the facility in relation to the wastewater generated and treated, inadequate onsite tests of basic wastewater parameters and inadequate employment of qualified and competent staff.
